# Surface Interactions between Bacterial Nanocellulose and B-Complex Vitamins

**DOI:** 10.3390/molecules25184041

**Published:** 2020-09-04

**Authors:** Diego Mauricio Sánchez-Osorno, Diego Gomez-Maldonado, Cristina Castro, María Soledad Peresin

**Affiliations:** 1 Facultad de Ingeniería agroindustrial, Universidad Pontificia Bolivariana, Circular 1°, No 70-01, Medellín 050031, Colombia; ingdiegosanchez@hotmail.com; 2 Forest Products Development Center, School of Forestry & Wildlife Sciences, Auburn University, 520 Devall Dr., Auburn, AL 36849, USA; dzg0023@auburn.edu; 3 Facultad de Ingeniería textil, Universidad Pontificia Bolivariana, Circular 1°, No 70-01, Medellín 050031, Colombia; cristina.castro@upb.edu.co

**Keywords:** bacterial nanocellulose, vitamins, QCM-D, adsorption, release

## Abstract

The interactions between films of bacterial nanocellulose (BNC) and B complex vitamins were studied using a Quartz Crystal Microbalance with Dissipation monitoring (QCM-D). Thin films of BNC were generated in situ by QCM-D, followed by real-time measurements of the vitamin adsorption. The desorption of vitamins was induced by rinsing the system using phosphate buffers at a pH of 2 and 6.5, emulating gastric conditions. Changes in frequency (which are proportional to changes in adsorbed mass, ∆m) detected by QCM-D were used to determine the amounts of vitamin adsorbed and released from the BNC film. Additionally, changes in dissipation (∆D) were proven to be useful in identifying the effects of the pH in both pristine cellulose films and films with vitamin pre-adsorbed, following its changes during release. The effects of pH on the morphology of the vitamin-BNC surfaces were also monitored by changes in rugosity from images obtained by atomic force microscopy (AFM). Based on this data, we propose a model for the binding phenomena, with the contraction on the relaxation of the cellulose film depending on pH, resulting in an efficient vitamin delivery process.

## 1. Introduction

Bacterial cellulose (BC) is a biosynthetic product of different *Gluconacetobacter* strains. This product was first discovered around 1886 by A. J. Brown, with the identification of the strain *Gluconacetobacter xylinus* [1,2]. Since its discovery, various research groups have been focused on developing different ways to optimize the production of the material, not only on a laboratory scale, but also at a semi-industrial level [3,4,5]. Because of the developments in research over the years, the knowledge base surrounding the formation and structure of BC has grown. The bacteria cells produce a so-called elementary cellulose fibril from their pores at the bacterium membrane. One of the most prominent characteristics of bacterial cellulose is a highly swollen three-dimensional (3D) network with a distinct tunnel, as well as its characteristic pore structure, and water content of up to 99% [6]. Nanofibrils in the range of 7–13 nm appear hydrated as a whole and are aggregated to flat microfibrils with a width of 70–150 nm, indicating that the water is outside of the crystalline cellulose structure [2]. Thus, bacterial nanocellulose offers distinctive properties based on the accessible hydrated nanofibrillated network. Besides its purity, a high degree of polymerization, and high crystallinity, this form of nanocellulose is especially distinguished by its biocompatibility and moldability during fermentation, as it is synthesized at the liquid-air interface. Furthermore, bacterial nanocellulose (BNC) fibers possess a small thermal expansion coefficient, similar to that of glass, with a high Young’s modulus (134 GPa), and a high tensile strength (2 GPa) [7,8].

Additional properties of bacterial nanocellulose include a high density, good shape retention, high water-binding capacity, and a higher surface area as compared to wood-based cellulose. These properties make BNC an excellent candidate to be used in areas, such as medicine and biomedical development [9,10,11], veterinary medicine [12,13], cosmetics [14,15], textile industry [10], mining and refinery [16], sewage purification, communications, paper industry [17], laboratories, electronics, energy [16,17,18,19]. Of all these industries, food and drug delivery systems have received particular attention in recent years [15,20]. In the case of food, BNC is edible, and it has been reported with an ability to be utilized as a multifunctional food ingredient, rheological enhancer [15,21], and a low-calorie food additive. Moreover, BNC has also been used in low cholesterol products [18], for thickening [11], and as a gelling and stabilizer agent [18,21,22]. Additionally, Müller et al. (2013) has reported BNC applicable as a drug delivery system for proteins using serum albumin as a model drug [23]. Moreover, the combination of BNC gel and amphiphilic block copolymer nanoparticles have been reported being used as a drug delivery system [24].

In most of the above-mentioned studies, the interaction of with BNC was described using a variety of analytical techniques, including spectrophotometry, Fourier-transform infrared spectroscopy (FTIR), near-infrared spectroscopy (NIRS), and nuclear magnetic resonance spectroscopy (NMR), mainly in the case of pharmacological applications [25]. However, a limited amount of research has been reported on these interactions using Quartz Crystal Microbalance (QCM-D). This method is a surface-sensitive technique that holds great potential to determine interactions, structural changes, and adsorption/desorption dynamics at the nanoscale by utilizing model surfaces. Conversely, QCM-D has been utilized to study the interaction of different compounds on spin-coated, model BNC surfaces [26,27].

The main advantage of utilizing QCM-D to study the interactions of active components like vitamins with BNC is the ability to follow in real-time the changes in dissipation and frequency of the thin films when vitamins are adsorbing or desorbing. These frequency changes can then be mathematically converted to mass changes, correlating them with the densities and viscosities of the used materials. Furthermore, the changes in dissipation also serve to indicate the physical changes in the film structure, as the response could indicate a more viscous or more plastic film during the changes in buffers and the interactions with the vitamins.

In this work, we investigate BNC’s potential to be used to adsorb different molecules, compounds, or active principles for food applications. In particular, we focus on the interactions of BNC and Vitamin B complex, due to the relevance of this vitamin for critical body functions like wound healing, muscle and nerve function, optimal muscle growth and development, as well as the production of red blood cells. The ingestion of vitamin B complex through normal processed food is not always possible due to vitamin degradation [28]. Vitamins of the B complex are highly degradable during cooking and processing because of factors as high temperature, oxygen, and light. In this work, we propose the utilization of BNC as a carrier of B complex vitamins, for improved vitamin stability and delivery through food products. The chemical composition of the vitamins was studied by Combs (1998) in the book “the vitamins, fundamental aspects in nutrition and health” [29]. However, to the best of our knowledge, no research has been conducted on the interactions of unmodified BNC and food components.

In this work, we utilize the piezoelectric sensing technique, QCM-D, to produce thin cellulose films in-situ and monitor the adsorption and desorption of B complex vitamins (B1, B2, B3, and B12) onto these surfaces at different pH values, emulating the human gastric system. We studied the morphology of surfaces during adsorption and desorption of the vitamins using atomic force microscopy (AFM).

## 2. Results and Discussion

### 2.1. Adsorption of Vitamins on Cellulose

**Figure 1:**
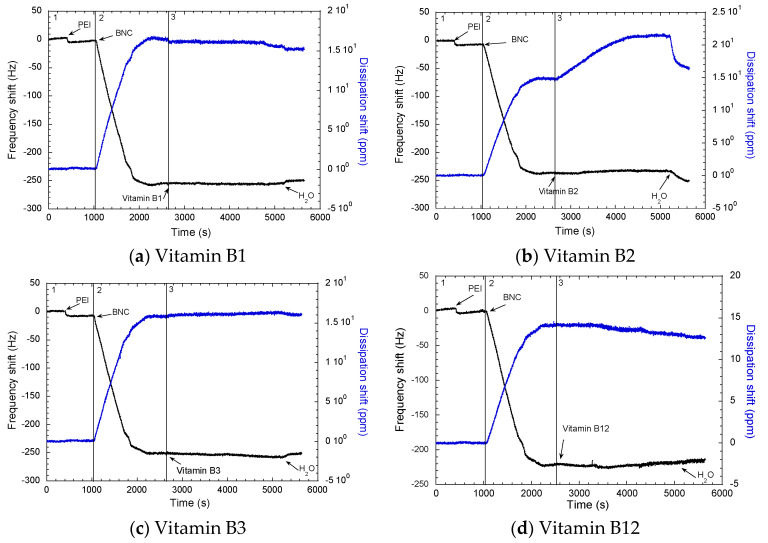
The shift in dissipation, ∆D, and frequency, ∆F from Quartz Crystal Microbalance (QCM-D) adsorption of vitamins: (**a**) B1, (**b**) B2, (**c**) B3, and (**d**) B12. ΔF = change in frequency (black line) and ΔD = dissipation (blue line). Adsorption zones: **a** (polyethylenimine, PEI), **b** (bacterial nanocellulose, BNC), and **c** (vitamin).

To understand the adsorption mechanism of the vitamins onto cellulose, molecular interaction should be analyzed. In general, the backbone of cellulose is formed by glucose monomers, each of them containing three hydroxyl groups. B complex vitamins (B1, B2, B3, and B12) have different chemical groups (hydroxyl or nitrogen groups) that have the possibility to participate in hydrogen bonds, which can be the postulated mechanism for the affinity of these vitamins with cellulose [30]. In acid solutions, the amino groups present in the vitamins are positively charged, showing good solubility in water. Nonetheless, in preliminary experiments, it was observed that these vitamins could be adsorbed on BNC surfaces between a pH of 6 to 7. Figure 1 shows the adsorption of PEI (polyethylenimine), BNC, and B complex vitamins in three observed zones.

In Figure 1, the first zone corresponds to the anchoring polymer (PEI) adsorption, the second zone corresponds to adsorption of the BNC, and the third zone is the adsorption of the vitamins onto the BNC surfaces. In the first zone, it is possible to note that PEI was adsorbed with a ΔF_PEI_ ranging between −18.5 Hz and −20 Hz. The adsorption of BNC on the surface ranges between 2788.84 ng/cm^2^ and 3423.50 ng/cm^2^, which corresponds to ΔF_BNC_ between −230 Hz to −260 Hz. Moreover, QCM-D experimentation indicated a small change in the frequency signal, followed by the adsorption leveling off after 30–40 min equilibration time. No desorption was evident after rinsing with ultrapure water.

**Figure 2:**
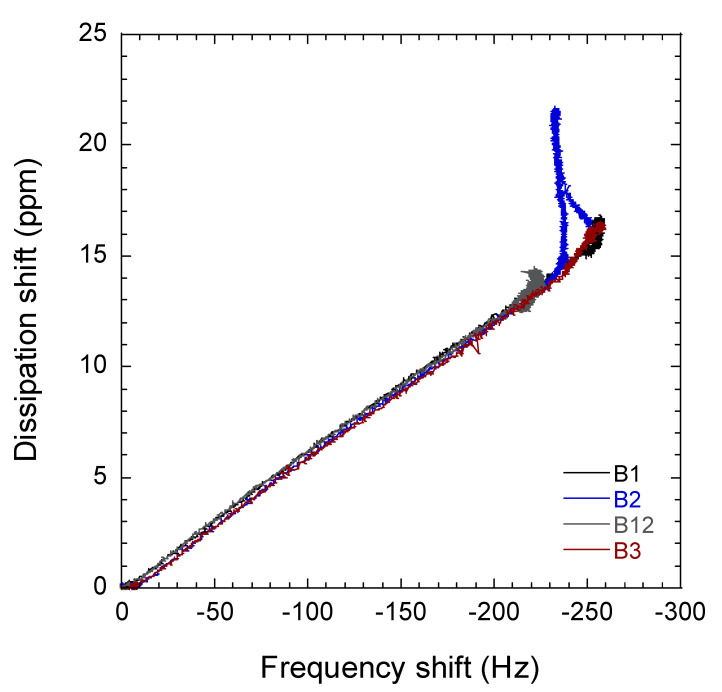
Dissipation shift–Frequency shift (ΔD−ΔF) plot of QCM-D for the generation of the BNC films and the adsorption of the vitamins.

Changes in QCM-D energy dissipation were plotted against the shift in frequency of the so-called ΔD−ΔF profiles (Figure 2). These profiles are important to identify the adsorption process of BNC in the surface of the sensor by PEI. Interestingly, the slopes of the ΔD−ΔF profiles in the second zone correspond to BNC adsorption. This was comparable in all cases, which confirms the adsorption mechanism of the BNC in PEI by in situ layer-by-layer built up was a proper method to form a monolayer film in the sensor [31]. It can be clearly observed that the intensity (changed from quantity) of adsorption (as determined by the maximum ΔF) was more limited for B12, followed by B2, B1, and B3 (ca. −226 Hz, −239 Hz, −257 Hz compared to ca. −257 Hz, respectively). Contrarily, the slopes of the ΔD−ΔF profiles in the third zone correspond to vitamin adsorption, indicating that a similar adsorption mechanism took place for vitamins B1, B3, and B12, though this mechanism is different for vitamin B2.

**Figure 3:**
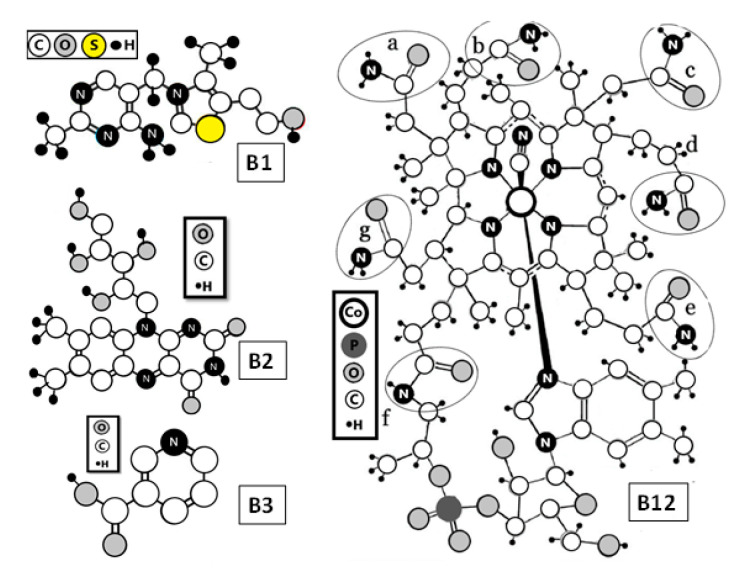
Molecular structure of vitamins B1, B2, B3, and B12.

Adsorption of vitamin B1, B2, and B12 were observed with little changes in ΔD (Table 1). Nonetheless, in the case of vitamin B2, a change is observed in ΔD when the vitamin is adsorbed from 140 ppm to 220 ppm. This change in ΔD suggests that the BNC surface is more viscoelastic upon B2 adsorption on the surface. Therefore, it is hypothesized that this viscoelastic change in BNC surface could be due to the action of ribitol group of riboflabin (B2). Moreover, ribitol groups contains four hydroxyl groups bonded together with high capacity to form H-bonds with BNC hydroxyl groups [32] (Figure 3). This might be due to the exerted attraction by ribitol to hydroxyl groups of BNC, which can cause a change in the structure of the cellulose network, as a higher attraction occurs between this group and BNC rather than the intermolecular H-bond. Based on ΔD changes, it is possible to see that BNC fibers are less tight after the interaction with ribitol groups.

Overall it is observed that all the vitamins are adsorbed in BNC; the amount absorbed is different for each vitamin. A greater quantity of vitamin B2 (riboflavin) is adsorbed per micrograms BNC, while the lowest quantity of vitamin absorbed per micrograms of BNC was the vitamin B3 (niacin).

Figure 3 shows the molecular structure of the vitamins (B1, B2, B3, and B12) in which the smaller size molecule corresponds to the vitamin B3, and the larger molecule size corresponds to the vitamin B12 with molecular weights of 123.11 g/mol and 1355.37 g/mol, respectively. In particular, it is interesting to note that the quantity of vitamin B3 absorbed on BNC is almost the same quantity of vitamin B12 absorbed, although the vitamin B12 molecule is 11 times larger than the vitamin B3 molecule. In the same way, it is observed that the quantity of vitamin B2 adsorbed was 2.5 times higher that vitamin B3 with values of 88.37 ng and 34.78 ng, respectively. In this case, the molecular weight of vitamin B2 is three times higher than vitamin B3. It is important to point out that the smaller molecular-sized vitamins are absorbed no more than the large molecular-sized vitamins. The adsorption of the vitamin is mediated by steric hidrance instead of molecular size. As explained below, in vitamin B2, the four hydroxyl groups of ribitol group are grouped together, obtaining a high capacity to form H-bond with BNC hydroxyl groups [32]. While for vitamin B2 the main interaction is initiated, due to OH groups, for vitamin B12, the interaction with BNC is proposed to be due to the special distribution of nitrogen and oxygen (Figure 3). These atoms are present on the side of each six-coordination cobalt complexes identified as a, b, c, d, e, f, and g that are located in different parts of B12 molecules. This distribution in vitamin B12 could promote the formation of hydrogen bonds, which is related to the adsorption and desorption process between B complex vitamins and BNC. Thus, suggesting the physisorption process as the dominant process between B vitamins and BNC [32]. In vitamin B1 and vitamin B3, the N or O groups are distributed around the molecule similar to vitamin B12, which is related to the quantity of vitamin adsorbed. In BNC, hydroxyl groups favor the formation of hydrogen bonds.

### 2.2. Desorption of Vitamins from Cellulose

**Figure 4:**
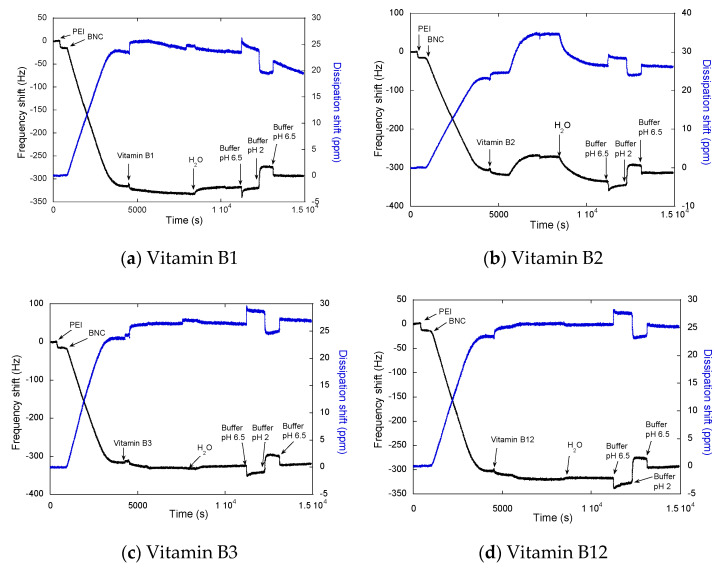
QCM-D plots for desorption of vitamins ((**a**) B1, (**b**) B2, (**c**) B3, and (**d**) B12)) by using pH 2 and pH 6.5. ΔF = change in frequency (black line) and ΔD = dissipation (blue line).

The influence of pH on the desorption process of the vitamins was studied through QCM-D by desorbing B complex vitamins (B1, B2, B3, and B12) from the BNC surfaces using two different pH values (2 and 6.5) and a phosphate buffer at 100 mM concentration (Figure 4) [33,34]. Those pH values were selected aiming to simulate the changes in pH experienced in the human digestive system. A common mechanism is observed in whole vitamin plots when buffers are added. When the buffer with a pH 6.5 is in contact with the surface, ΔF is more negative from −350Hz approx. to −340 Hz approx., indicating an augmentation in the mass related to the adsorption of the salt and water as an effect of the change of the medium. In contrast, when pH 2 is added, it is observed that ΔF is less negative, indicating mass reduction with values from −340 Hz approx. to −310 Hz approx. This is indicative of reversible adsorption that occurred even when electrostatic repulsion was favored, that is, when the charges for vitamin and cellulose were of the same sign. The effect of the pH in both ΔF and ΔD is expected, due to pH affecting the dissociation of acid groups (interaction between cellulose surface). A pH of 6.5 decreases ΔF and increases ΔD, which could be explained by the adsorption of water and buffer salts, while pH is opening the cellulose network and increasing viscoelasticity. The opposite effect is observed with a pH of 2, where a ΔF increase and a ΔD decrease showed that the BNC network would have high rigidity, and it is possible to observe some release of vitamins and adsorbed water from the surface. This effect was observed for some authors, showing that when the pH increases, the cellulose films swelled considerably [35,36]. This effect was observed in pH-responsive bacterial cellulose/acrylic acid hydrogels at a pH of 2 when the swelling rate slightly increased from 8 h at a pH of 2 to 48 h at a pH of 7. Nonetheless, the sensitivity of the response of the hydrogels to changes in pH (from pH 2 to pH 7) was likely due to the hydrophilicity of the carboxylic group in the hydrogel structure [35]. The results of this research show that bacterial cellulose is pH sensitive and changes by itself, even without crosslinking. Additionally, the solubility of cellulose in aqueous alkali solutions correlates well with the decrease of hydrogen bonding, this structural parameter is referred to as “the degree of break-down of intramolecular hydrogen bonds (O3–H⋯O5) of cellulose [37,38]. Plots of Figure 4 show that at a pH of 2 there is no vitamin delivered. When the pH is changed to 7, the structure of the BNC web is opened, allowing for delivery [39]. This also confirms that we can modulate the release of the vitamins with a change in the pH conditions, which is related to the trajectory that this would follow in normal digestion.

### 2.3. Surface Morphological Analysis (AFM)

**Figure 5:**
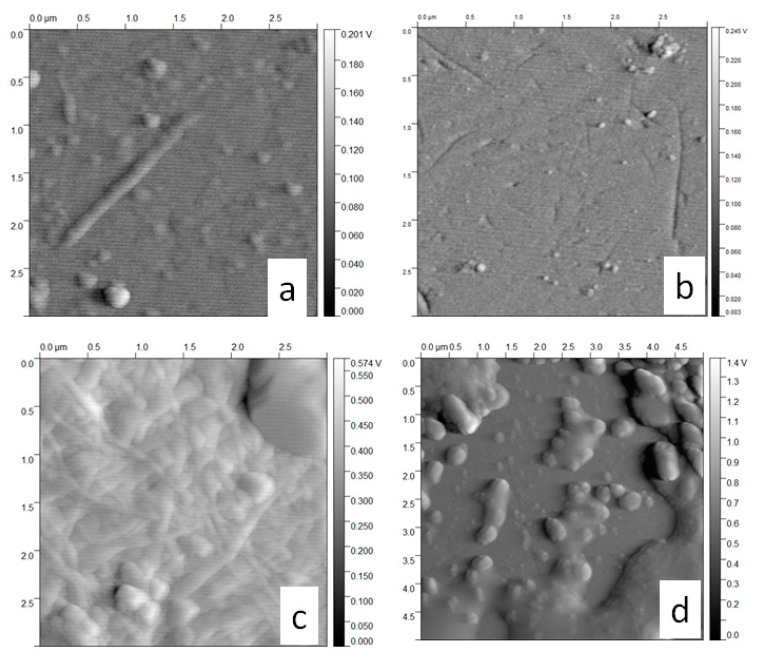
Interaction vitamin B12 vs. BNC AFM images. Adsorption: (**a**) BNC Adsorption; (**b**) BNC Desorption; (**c**) B12 Adsorption; (**d**) B12 Desorption.

To better understand the changes of the BNC thin films after interacting with the vitamins and different pHs, AFM images of the surfaces before and after the adsorption of vitamins were taken and are shown in Figure 5. The general observation was that the surface of BNC changes with both adsorption and desorption of B complex vitamins. For a better comparison, root mean squared roughness (RMS), skewness (Rsk), or degree of bias of the roughness shape, and kurtosis (Rku), or measure of the sharpness of the roughness profile (Table 2), are used to indicate the topographical variation [40]. In this sense, it is possible to observe that all the vitamins of this experiment, as well as both process adsorption and desorption, produce topographical variations in BNC surface. However, the highest variation was observed with adsorption and desorption of vitamin B12. Skewness for BNC was −0.08, indicating a surface with imperfections and space between the fibrils that formed this surface. When the different vitamins are adsorbed onto BNC, the skewness values changed. For vitamin B1, skewness was 0.25; this positive value shows the formation of protrusions instead of holes [41,42]. This could be an indicator that vitamin B1 diffuses into the spaces in between the fibrils of the BNC surface. The skewness parameter for vitamin B2 is −0.03 instead of −0.08 in BNC also indicate a diffusion of the vitamin into the surface, even if lower compared to vitamin B1. Conversely, with the adsorption of vitamins B3 and B12, the surface shows profiles with peaks removed, or deep scratches, when compared with the BNC surface. The higher separation between the fibrils was noted in the B12 surface with the skewness value of −0.14, followed by B3 surface skewness of −0.39, which could be related to a stronger pull of these molecules through the surface. [41,42]. The kurtosis parameter has a considerable change in BNC between both adsorption and desorption, showing that after a pH of 2 and 7, high peaks are formed. Changes in the BNC fibril network above are explained and are coherent with kurtosis parameters. The kurtosis value of the BNC surface was 2.88, but this value is lower when vitamin B12 is adsorbed, with 2.82. B1, B2, and B3 adsorption kurtosis values higher than BNC kurtosis (Table 2) [43]. With the classification of kurtosis as leptokurtic with values higher than 3, and platykurtoic with values lower than 3 [43], we observe that adsorption for vitamins B1, B2, and B3 could be considered leptokurtic and relatively has many high peaks and low valleys. Furthermore, the adsorption kurtosis value for B12 is considered as platykurtoic and has relatively few high peaks and low valleys. For desorption, it was found that kurtosis of vitamins is always lower when values are compared with BNC (after) kurtosis. After the desorption of vitamins, all the surfaces were classified as leptokurtic, in the following order: B1 < B12 < B3 < B2 < BNC.

The above AFM results and the ∆F from QCM-D analysis reveal the adsorption of vitamins onto the surface of BNC. Values obtained for roughness parameters, shown in Table 2, can be considered characteristics of each sample [40]. Additionally, it is observed that after desorption, the BNC parameters do not return to initial values (Table 2), indicating that the vitamin adsorption on the BNC surface is irreversible to a certain extent. This change in the morphology could be due, not only to the release of vitamins, but to the effect of the buffers with a pH 2 and pH 6.5, allowing the relaxation or contraction of BNC as explained in QCM-D analysis. The structural changes observed in BNC by AFM are correlated with film swelling observed in QCM-D as the repulsion between cellulose surfaces increases when the pH is raised. It is believed that this increase is due to the swelling of the cellulose films, and it is possible that this swelling is due to the charging of the cellulose layer [36].

When comparing the BNC surface (after experimentation) with the surface during B1 adsorption, it was observed that Rsk increases, and Rku show higher (and relatively more defined peaks) which could be an indicator that B1 is adsorbed in the holes and peaks of the biomaterial BNC. On the other hand, comparing the adsorption and desorption BNC surfaces, the desorption process shows fewer holes (skewness), and less defined peaks (kurtosis) than in adsorption. Furthermore, B2 adsorption is similar to B1 adsorption, where Rsk increase, and Rku is higher (with defined peaks), indicating that B2 is adsorbed in the holes and peaks of BNC. In the desorption process exhibited fewer holes (skewness), and less defined peaks (kurtosis) are observed. This suggests that the release process of the vitamins from the BNC surface begins at the peaks and not the holes. Even when B2 presented an adsorption similar to B1, the images shown a higher Rsk and Rku increase for B2, these defined peaks suggest that B2 is adsorbed both on the energetic points found at the peaks and on the less energetic valleys of BNC. Therefore, in the desorption process, fewer holes (skewness), and less defined peaks (kurtosis) are observed. Additionally, we think that the adsorption of B3 primarily occurs near the peaks and the baseline, with minimal adsorption in the holes. The desorption process of B3 shows more valleys (Rsk), as well as pronounced and defined peaks, indicating that desorption of vitamin B3 is a similar mechanism of attrition where there are losses of mass from the side of the peaks. The adsorption process of vitamin B12 produces deep holes in BNC surface, but after desorption, the skewness is almost the same. AFM images of the bacterial nanocellulose (BNC) when used to adsorb vitamins B1, B2, B3, and B12 and after treatment with different pH could be seen in Supplementary Materials (Table S1).

## 3. Materials and Methods

### 3.1. Materials

In this study, agro-industrial waste from fruits: Mangifera indica (mango), Malus domestica Borkh (apple), Ananas sativus (pineapple), carica papaya (papaya), and musa paradisiac (banana) were obtained from the Medellin archdiocesan food bank. These fruits were washed, crushed, squeezed; then residues and juice were separated.

The blend was made in a SKYMSEM LAR-04MB for six minutes total, and filtration was done through nylon strainers of different pore diameters. The obtained juice was standardized to 4°brix by adding water and modified to a pH of 3.5 by acetic acid. The aim of using fruit juice was as a carbohydrate source (culture medium) for the incubation of bacteria, which was sterilized at 15 psi for 20 min before inoculation.

Bacterial nanocellulose membranes were obtained by using the *Komagataeibacter medellinensis* strain a cultivated in before-mentioned homemade fruits juice. Bacterial nanocellulose aqueous dispersions were prepared as follows: Bleached bacterial cellulose membranes were mechanically treated using a Masuko Supermass Colloider (Japan) and stored at 4 °C for further use. Then, a suspension of cellulose (0.5% *w*/*v*) was stirred at 100 rpm for 15 min, and the resulting BNC suspension was centrifuged at 600 rpm for 45 min. Cellulose thin films were prepared in situ in the QCM-D chamber, using the supernatant of BNC suspension. Vitamins B1, B2, B3, and B12 were purchased from Sigma-Aldrich, (Lot: LC08344V, St. Louis, MO, USA). Polyethylenimide, branched LOT # MKCB1222V average Mw ca. 25,000 by LS, average Mn ca. 10,000 by GPC (Aldrich, St. Louis, MO, USA). Aqueous vitamin solutions were prepared based on the maximum vitamin solubility with the following concentrations: B1 (61 mg/100 mL), B2 (107 mg/100 mL), B3 (132 mg/100 mL), B12 (91 mg/100 mL). The water used was deionized and purified with a Thermo Scientific Barnstead Nanopure (18.2 MΩ cm), 100 mM phosphate buffer pH 6.5 and pH 2 were prepared prior to each experiment.

### 3.2. Quartz Crystal Microbalance with Dissipation Monitoring (QCM-D)

All the experiments were performed on a Q-Sense analyzer (Biolin Scientific. Västra Frölunda, Sweden). These measurements were performed on silicon dioxide (SiO_2_) coated QCM-D sensors that were purchased from Biolin Scientific (Västra Frölunda, Sweden). Before each test, the sensors were cleaned by standard chemical and UV-ozone plasma treatments. In the plasma treatment, the incident UV light oxidizes any spurious adsorbed organic matter that could remain on the surface of the sensor and also activates silanol groups required in later coating steps. Thereafter, QCM-D measurements were performed at the resonance frequency of 5 MHz, and the overtones 3, 5, 7, 9, 11, and 13 of the base frequency were recorded. To simplify the reading, only data collected from the third overtone 3 is presented. Thin films of cellulose were generated as model substrates prepared in situ by flowing the BCN supernatant suspension through the QCM-D chamber. Frequency and dissipation shifts were modeled to obtain mass changes with DFind Software from Biolin Scientific (Västra Frölunda, Sweden) using density values of 1.2 g cm^−3^ for cellulose and 0.5, 0.55, 0.1, and 0.33 g cm^−3^ for vitamins B1, B12, B2, and B3, respectively; density of buffers changed accordingly to pH, being 1.007 g cm^−3^ for pH 2 at 25 °C and 1.080 g cm^−3^ for pH 6.5 at 25 °C; finally, water density was 0.997 g cm^−3^. A Broadfit model was used with frequency dependency on viscosity and elastic modulus.

#### 3.2.1. Adsorption of Vitamin B Complex to BNC

In a typical experiment, mili-Q water was injected into the QCM-D flow module (chamber) assembled with a cleaned-QCM silica sensor. The sensor remained in contact with the background water for several minutes to allow equilibrium to be attained with the medium in order to collect the base QCM-D signal, or baseline, at a flow rate of 150 µL/min at 25 °C. Following this, 0.1% (*m*/*v*) polyethylenimine (PEI) solution was continuously introduced into the cell with a peristaltic pump until equilibrium was reached; the aim of PEI is to serve as an anchoring polymer, inducing the attachment of BNC to the sensor. Next, Mili-Q water flow was used to rinse the non-attached PEI molecules. Utilizing the same flow conditions, BNC solution was introduced into the cell until equilibrium was reached, in order to form the BNC thin film on the QCM-D sensor. For adsorption of vitamins on BNC, a third solution containing the respective vitamins in mili-Q water was flown into the chamber until equilibrium is reached. A rinsing step using mili-Q water was performed after each step.

#### 3.2.2. Desorption of Vitamin B Complex from BNC

For desorption studies, after the adsorption of the vitamins on BCN surfaces, the medium was changed to phosphate buffer (pH = 6.5). Upon system stabilization, a new solution containing phosphate buffer at a pH of 2 was poured through the chamber until equilibrium was reached, followed by a new flow of phosphate buffer solution at a pH of 6.5.

For each experiment, each of the solutions was injected only after ensuring that the drift of the third overtone frequency ($\Delta f_{3}$) for the cellulose-coated sensor in the background was lower than 2 Hzh^−1^. This ensures a full stabilization of the film in each condition (fully swollen film and stress-free system). All measurements were performed at least twice to ensure reproducibility.

### 3.3. Cellulose Morphological Characterization by Atomic Force Microscopy (AFM)

The thin cellulose films were characterized in terms of material distribution, surface roughness, and topography using an AFM Dimension 3100 (Santa Clara CA, USA) prior to and after the interactions with vitamins. Amplitude images were obtained with tapping mode at 2.35 Hz of amplitude velocity with a Nano World (Innovative Technologies, CH-2000 Neuchâtel, Switzerland) FM 20 silicon SPM-sensor. Roughness analysis and images processing were performed by Gwyddion software 2.49 (SourceForge).

## 4. Conclusions

In conclusion, this work confirms that BNC interacts with vitamins of the B complex, mainly by hydrogen bonds. Even if whole vitamins of the B complexes are adsorbed by the BNC surface, and each vitamin is adsorbed and desorbed in a different way. It is apparent that pH is an important factor that can be used to determine the release profile of each vitamin. At a low pH, the BNC network is rigid, while at a pH around 7, the BNC network begins to open, and adsorption or desorption is facilitated. The reaction of BNC at different pHs presents the possibility for the use of BNC in an array of applications, such as in nanofood, to optimize the delivery at structures where the vitamins can be better utilized. Furthermore, BNC expresses a property important for drug delivery systems, as we demonstrated that changes in pH can tune the release of the carrier molecules. This capability would also be important for food applications, since the stomach presents lower pH that would determine the possibility of the release inside of it; also in medical and antibiotic applications as bacteria growth has an impact on the pH of its surroundings, which opens the potential of generating smart materials based on BNC.

## 5. Patents

UNIVERSIDAD PONTIFICIA BOLIVARIANA and CORPORACIÓN UNIVERSITARIA LASALLISTA. “Sistema de protección y liberación controlada de micronutrientes”. Colombian Patent No. NC2020/0003001 filed 13 March 2020.

## Figures and Tables

**Table 1:** Quantifications of changes in frequency (ΔF), changes in dissipation (ΔD) and mass calculated (Mass); Adsorption = ads; Desorption = des.

Vitamin	∆F_ads [Hz]	∆D_ads [ppm]	∆F_des [Hz]	∆D_des [ppm]	Mass_ads [ng/cm^2^]	Mass_des [ng/cm^2^]	ng vit/µg BNC
B1	−16.7	1.14	25	−3.89	132.76 ± 12.41	−355.38 ± 21.55	43.5 ± 9.03
B2	32.3	−8.34	21.78	−0.69	302.55 ± 12.86	−16.95 ± 11.54	88.37 ± 13.2
B3	−16.2	2.6	4.62	0.55	109.56 ± 8.08	−124.57 ± 10.11	34.78 ± 7.46
B12	−16	2	23.1	−0.6	182.43 ± 15.73	436.23 ± 29.37	37.52 ± 4.09

**Table 2:** Roughness parameters obtained by atomic force microscopy (AFM) analysis of BNC and B complex vitamins surfaces.

Parameter		BNC		B1		B2		B3		B12	
		Before	After	Before	After	Before	After	Before	After	Before	After
RSM (Rq) (nm)	Mean	1.16	1.21	1.50	1.89	2.20	1.11	2.53	2.80	3.10	3.28
	std	0.11	0.05	0.08	0.07	0.41	0.10	0.08	0.16	0.08	0.23
Skewness (Rsk)(–)	Mean	−0.08	0.20	0.25	0.13	−0.03	−0.22	−0.39	−0.30	−0.14	−0.13
	std	0.01	0.12	0.19	0.11	0.09	0.12	0.25	0.07	0.02	0.02
Kurtosis (Rku) (–)	Mean	2.88	4.94	3.39	3.14	4.08	4.16	3.49	3.66	2.82	3.30
	std	0.06	0.59	0.13	0.47	0.47	0.17	0.60	0.11	0.12	0.08

(–): no unit; (nm): nanometer.

## Supplementary Materials

Table S1. Height AFM images of the bacterial nanocellulose (BNC) when used to adsorb vitamins B1, B2, B3, and B12 and after treatment with different pH.
