# Supplementary material for: Surface Interactions between Bacterial Nanocellulose and B-Complex Vitamins
Source: Molecules. 2020 Sep 4;25(18):4041. doi: 10.3390/molecules25184041 (PMC7571027; doi:10.3390/molecules25184041)
Supplement: Supplementary file 1 [file molecules-25-04041-s001.pdf]

Supplementary information

Surface Interactions between bacterial nanocellulose and B-complex vitamins

Diego Mauricio Sánchez-Osorno [1], Diego Gomez-Maldonado [2], Cristina Castro [3], and María Soledad Peresin [2,*]

[1] Facultad de Ingeniería agroindustrial; Universidad Pontificia Bolivariana; Circular 1°, No 70-01, 050031 Medellín, Colombia; ingdiegosanchez@hotmail.com
[2] Forest Products Development Center, School of Forestry & Wildlife Sciences, Auburn University, 520 Devall Dr., Auburn, AL 36849, USA; dzg0023@auburn.edu
[3] Facultad de Ingeniería textil; Universidad Pontificia Bolivariana; Circular 1°, No 70-01, 050031 Medellín, Colombia; cristina.castro@upb.edu.co
* Correspondence: soledad.peresin@auburn.edu; Tel.: +1-334-559-1143

Table S1. Height AFM images of the bacterial nanocellulose (BNC) when used to adsorb vitamins B1, B2, B3 and B12 and after treatment with different pH.

<table>
<tr><td>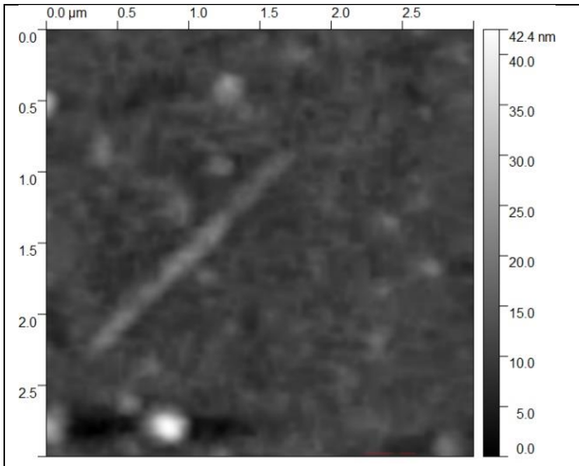
</td><td>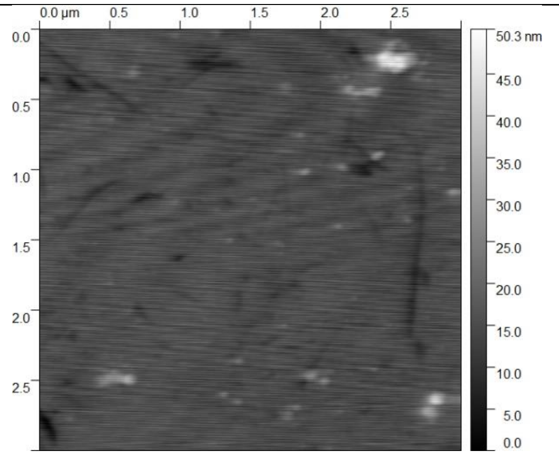
</td></tr>
<tr><td>BNC adsorption</td><td>BNC desorption</td></tr>
</table>

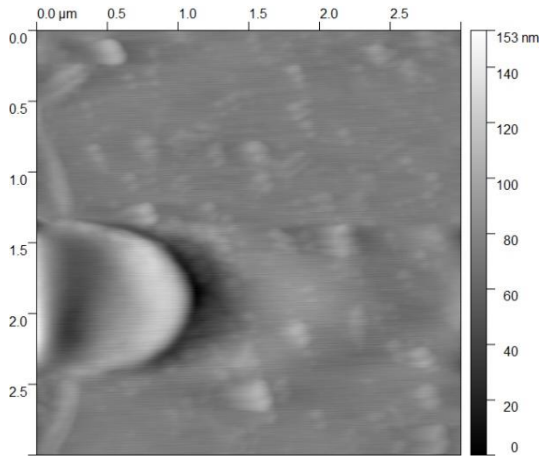	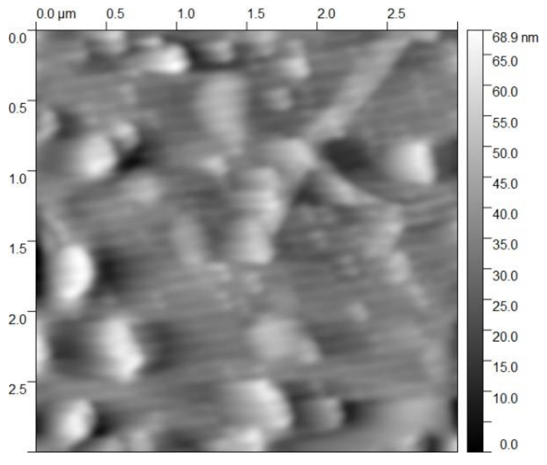
Vitamin B1 adsorption	Vitamin B1 desorption
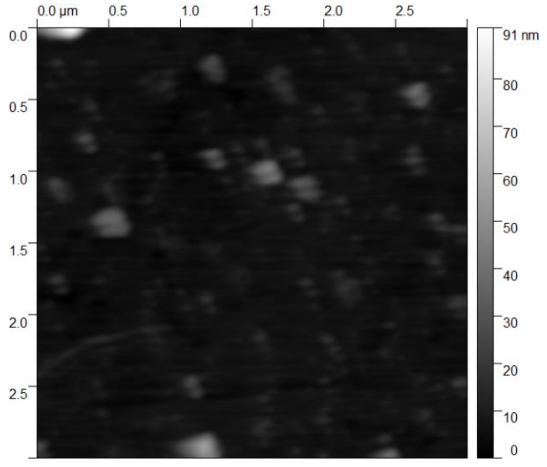	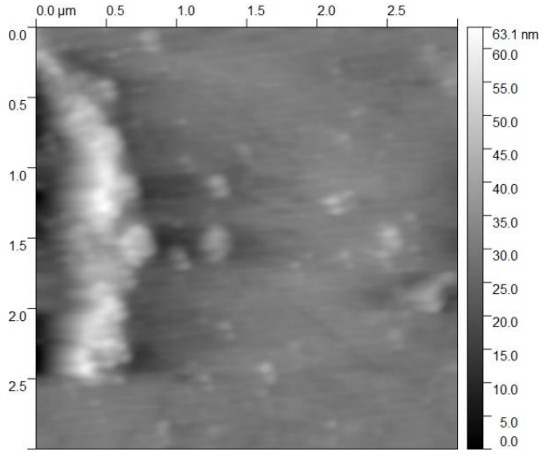
Vitamin B2 adsorption	Vitamin B2 desorption
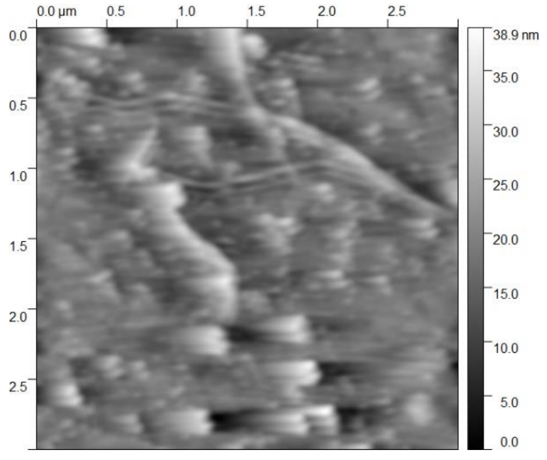	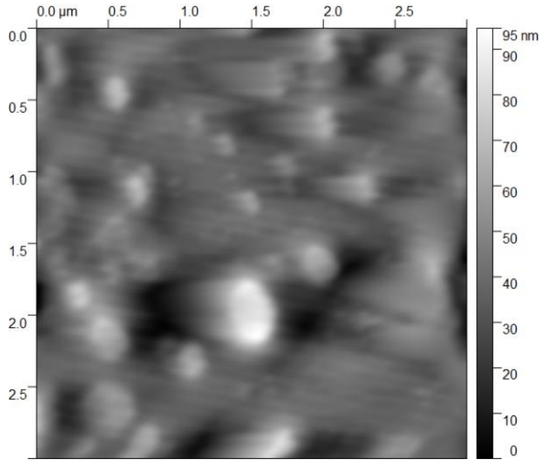

<table>
<tr><td>Vitamin B3 adsorption</td><td>Vitamin B3 desorption</td></tr>
<tr><td>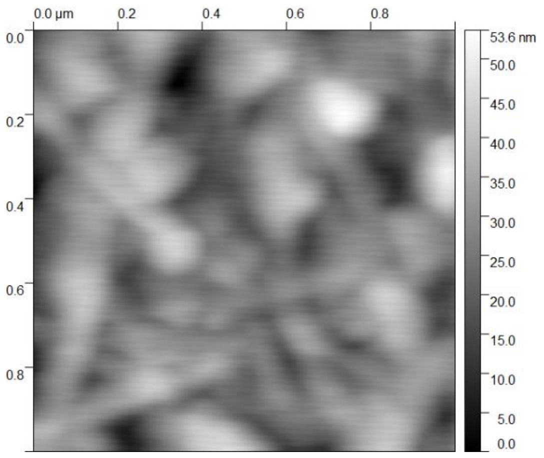
</td><td>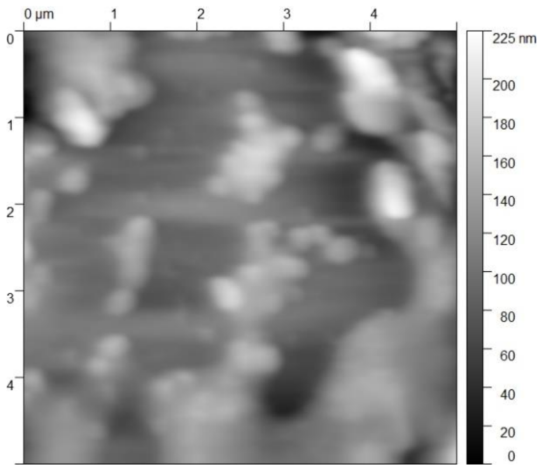
</td></tr>
<tr><td>Vitamin B12 adsorption</td><td>Vitamin B12 desorption</td></tr>
</table>
